# Phytochemical Analysis, Network Pharmacology and in Silico Investigations on *Anacamptis pyramidalis* Tuber Extracts

**DOI:** 10.3390/molecules25102422

**Published:** 2020-05-22

**Authors:** Mohamad Fawzi Mahomoodally, Marie Carene Nancy Picot-Allain, Gokhan Zengin, Eulogio J. Llorent-Martínez, Hassan H. Abdullah, Gunes Ak, Ismail Senkardes, Annalisa Chiavaroli, Luigi Menghini, Lucia Recinella, Luigi Brunetti, Sheila Leone, Giustino Orlando, Claudio Ferrante

**Affiliations:** 1 Department for Management of Science and Technology Development, Ton Duc Thang University, Ho Chi Minh City 758307, Vietnam; mohamad.fawzi.mahomoodally@tdtu.edu.vn; 2 Faculty of Applied Sciences, Ton Duc Thang University, Ho Chi Minh City 758307, Vietnam; 3 Department of Health Sciences, Faculty of Science, University of Mauritius, Réduit 230, Mauritius; picotcarene@yahoo.com; 4 Department of Biology, Science Faculty, Selcuk University, Campus, Konya 42130, Turkey; akguneselcuk@gmail.com; 5 Department of Physical and Analytical Chemistry, University of Jaén, Campus Las Lagunillas S/N, E-23071 Jaén, Spain; ellorent@ujaen.es; 6 Chemistry Department, College of Education, Salahaddin University-Erbil, Erbil 44001, Iraq; hassan.abdallah@su.edu.krd; 7 Department of Pharmaceutical Botany, Faculty of Pharmacy, Marmara University, Istanbul 34668, Turkey; isenkardes@marmara.edu.tr; 8 Department of Pharmacy, “G. d’Annunzio” University Chieti-Pescara, 66100 Chieti, Italy; annalisa.chiavaroli@unich.it (A.C.); lucia.recinella@unich.it (L.R.); luigi.brunetti@unich.it (L.B.); sheila.leone@unich.it (S.L.); giustino.orlando@unich.it (G.O.); claudio.ferrante@unich.it (C.F.)

**Keywords:** *Anacamptis pyramidalis*, antioxidant, enzyme inhibition, phytochemical fingerprint, network pharmacology, docking study

## Abstract

*Anacamptis pyramidalis* (L.) Rich. forms part of the Orchidaceae family that is highly valued for its horticultural as well as therapeutic benefits. The present study set out to investigate the inhibitory activity of *A. pyramidalis* tubers against key biological targets for the management of type 2 diabetes, Alzheimer disease, and skin hyperpigmentation. In addition, the antioxidant potential of the extracts was also assessed using multiple methods. The detailed phytochemical profiles of the extracts were determined using high-performance liquid chromatography. Based on qualitative phytochemical fingerprint, a network pharmacology analysis was conducted as well. Parishin was identified from the water extract only, whereas gastrodin and caffeic acid derivatives were present in the methanol extract. The methanol extract exhibited high inhibitory activity against tyrosinase (69.69 mg kojic acid equivalent/g extract), α-amylase (15.76 mg acarbose equivalent/g extract), and α-glucosidase (20.07 mg acarbose equivalent/g extract). Similarly, the methanol extract showed highest antioxidant potential (22.12, 44.23, 45.56, and 29.38 mg Trolox equivalent/g extract, for 2,2-diphenyl-1-picrylhydrazyl (DPPH), 2,2′-azino-bis(3-ethylbenzothiazoline-6-sulfonic acid) (ABTS), CUPric Reducing Antioxidant Capacity (CUPRAC), and Ferric Reducing Antioxidant Power (FRAP) assays, respectively). Finally, the results of network pharmacology analysis, besides corroborating traditional uses of plant extracts in the management of cold and flu, confirmed a direct involvement of identified phytochemicals in the observed enzyme inhibitory effects, especially against tyrosinase, α-amylase, and α-glucosidase. Furthermore, based on the results of both colorimetric assays and network pharmacology analysis related to the activity of *A. pyramidalis* extracts and identified phytocompounds on enzymes involved in type 2 diabetes, a docking study was conducted in order to investigate the putative interactions of oxo-dihydroxy octadecenoic acid trihydroxy octadecenoic acid against aldose reductase, peroxisome proliferator-activated receptor (PPAR)-α, dipeptidyl peptidase (DPP)-IV, and α-glucosidase. Docking analysis suggested the inhibitory activity of these compounds against the aforementioned enzymes, with a better inhibitory profile shown by oxo-dihydroxy octadecenoic acid. Overall, the present findings supported the rationale for the use of *A. pyramidalis* as source of bioactive metabolites and highlight, today more than ever, for the strong necessity of linkage strategy between wild resource valorization and conservation policy.

## 1. Introduction

A resurgence in general public interest towards natural products has been observed over the past decades. Indeed, natural products have continuously been the major source of therapeutics for the management of multiple human ailments since time immemorial. To date, the majority of the world’s population still relies on plants for primary health care [1]. Plants, particularly those used in traditional medicine, are the mainstay of early drug discovery. In fact, the identification of hits using high-throughput techniques underpins the optimization of lead compounds, which will be eventually used for the development of new drugs.

The Orchidaceae family comprises approximately 20,000 species divided in 796 genera distributed worldwide [2]. The diverse, colorful, and fragrant blooms of orchids make them highly praised horticultural species. In addition, orchidaceae species are highly valuable medicinal plants which have a very long history of use in folk medicine among different cultures across the world. For instance, the stem infusion of *Ansellia africana* Lindl., also known as the “leopard orchid”, endemic to Africa, was used as antidote against nightmares by the Zulu. An infusion of the leaves and stems was used to treat madness in Zambia. In Zimbabwe, *A. africana* was used as aphrodisiac [3]. *Dendrobium nobile* Lindl., known for its violet flowers, is one of the most important medicinal orchids referenced in pharmacopeias worldwide [4]. Chinsamy and colleagues [2] published a comprehensive article about the different orchid species used in traditional medicine in South Africa. In Turkey, one to two cups per day of the dried powdered tubers of *Anacamptis pyramidalis* (L.) Rich. infused with milk is taken for one to two weeks against cold and flu, as body warmer, as a vasodilator, and as tonic [5]. Dried powdered tubers of *A. pyramidalis* are used as a spice and to manage miscarriage [6]. *A. pyramidalis* tubers are also used in the preparation of “salep”, a traditional beverage also used as stabilizer in the preparation of ice cream [7]. The large use is documented in reports of tons of plant material trade originating from wild collection, during the last centuries [8]. To this practice, as well as to ethnomedicinal uses, are related the effects of many orchids within the endangered plants, and many specific conservation policies were activated. In Europe, many orchids are protected and the commercial use is prohibited [9].

In some countries, an internal trade of orchid tubers for medicinal, food, and beverage use is regulated to manage the potential risk related to the sustainability of the practice and to guarantee the genetic resource preservation. This practice, coupled with a relevant illegal collection of tubers for international trade, is considered the main reason for orchid population decline that in some cases consists of high risk of extinction [8,10,11].

Following a thorough literature search, a paucity of scientific information regarding the biological activity of *A. pyramidalis* tubers was noted. Therefore, this study was designed to establish the antioxidant, enzyme inhibitory (cholinesterase, tyrosinase, amylase, and glucosidase), and phytochemical profiles of the methanol and water extracts of *A. pyramidalis* tubers. The enzymes targeted are linked to global health problems including Alzheimer disease, hyperpigmentation, and diabetes mellitus. From this point, we searched for new raw materials for the management of these diseases. Additionally, according to the qualitative phytochemical fingerprint, a network pharmacology analysis was conducted, with the aim to elucidate the putative target proteins underlying the observed biological effects, whereas a docking analysis was conducted to confirm the interactions between selected phytochemicals and enzymes underlying the observed biological effects. It is expected that data generated from this investigation will provide valuable insights on the pharmacological potential of *A. pyramidalis* tubers, thus validating the traditional uses of this plant and improving the local product chain, also in terms of sustainability.

## 2. Results and Discussion

### 2.1. Phytochemical Profile

The standard assessment of bioactive compounds, in terms of phenolic and flavonoid contents is presented in Table 1. From data presented, *A. pyramidalis* tuber possessed low concentration of flavonoids. The phenolic contents of methanol and water extracts were not significantly (*p* > 0.05) different, thereby suggesting that solvents used were equally potent in extracting phenols from *A. pyramidalis* tuber. The major shortcoming of standard spectrophotometric methods in the determination of bioactive contents is the interference of other nonphenolic compounds, such as vitamins, which might yield inaccurate data. The use of high-performance liquid chromatography coupled to mass spectrometry detection in the field of phytochemistry provides explicit and detailed profiles of plant extracts, which might be crucial in understanding interaction with specific proteins.

**Figure 1:**
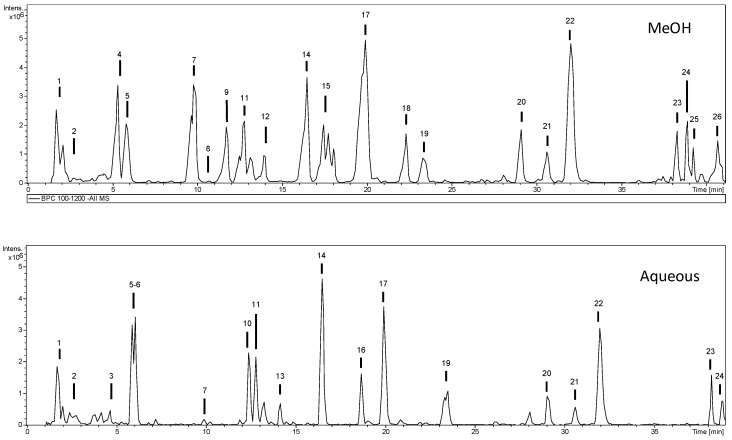
Base peak chromatogram of the methanol and aqueous extracts of *A. pyramidalis*.

To our best knowledge, this is the first report on the phytochemical profile of *A. pyramidalis*. The most abundant compounds were parishin and gastrodin derivatives, as well as a caffeic acid derivative. Similar fragmentation profiles have been reported in *Gastrodia elata*, also from the Orchidaceae family. Identification of compounds was performed by mass spectrometry (MS^n^)in negative mode. The identification was performed by comparison with analytical standards, when available, and by comparison of mass fragmentation profiles (MS/MS) with data from scientific literature. Compounds were numbered according to their retention times, keeping the same numeration in methanol and water extracts. Table 2 shows the characterization of compounds, whereas chromatograms are shown in Figure 1.

The fragmentation of compound **1** corresponded to a disaccharide formed by two hexose units: Loss of 162 daltons (Da) and fragment ions at *m/z* 179 and 161. It was present in both aqueous and methanolic extracts.

Compound **2** was identified as citric acid due to its [M − H]^−^ at *m/z* 191 and fragment ions at *m/z* 173 and 111 (comparison with an analytical standard). Several citric acid glycosides were also characterized in *A. pyramidalis* extracts. Compounds **3** and **6** exhibited deprotonated molecular ions at *m/z* 459 and fragment ions at *m/z* 173 and 111 (typical of citric acid). This fragmentation has been previously reported for parishin G, 2-[4-*O*-(β-d-glucopyranosyl)benzyl] citrate, in *G. elata* [12]. Compounds **10** and **13** exhibited the same fragmentation patterns; they were characterized as parishin B and parishin C, respectively, taking into account their elution order [13]. Compound **16**, with [M − H]^−^ at *m/z* 995, was identified as parishin by comparison with bibliographic data [13].

Compound **8** corresponded to the formate adduct of roseoside (vomifoliol glucoside or drovomifoliol-*O*-β-d-glucopyranoside) [14]. This compound has not been previously reported in species of the Orchidaceae family.

Several gastrodin derivatives, most of them present only in the methanolic extract, were tentatively characterized. Compounds **9** and **12** suffered the neutral loss of 286 Da (gastrodin), whereas compounds **11**, **14**, and **17** exhibited fragment ions at *m/z* 285, 161, and 123, characteristic of gastrodin [13].

Compounds **15**, **19**, and **20** were tentatively characterized as dihydroxybenzoic acid derivatives due to the 153→109 fragmentation. Compound **22** was characterized as a caffeic acid derivative due to the fragment ions at *m/z* 179 and 135 (comparison with an analytical standard).

Two acacetin derivatives were found in both methanol and aqueous extracts: Compounds **23** and **24**. Both of them presented the aglycone acacetin at *m/z* 283, which was identified by its fragment ion at *m/z* 268.

Compounds **25** and **26**, only present in the aqueous extract, were characterized as the oxylipins oxo-dihydroxy-octadecenoic acid and trihidroxy-octadecenoic acid [15], bioactive compounds that are produced in the oxidative metabolism of polyunsaturated fatty acids.

A relative semi-quantification was performed to check for the most abundant compounds. It was done by measuring peak areas of each compound in MS mode using the extracted ion chromatograms, with precursor ion [M − H]^−^. The relative percentage of each compound was calculated and is shown in Table 3, in which the heat map highlights the most abundant compounds (the darker the color, the higher the concentration). It can be observed that gastrodin derivatives (38.5%) and a caffeic acid derivative (compound **22**, 16%) were the most abundant compounds in methanol extract, whereas the aqueous extract was also rich in gastrodin derivatives (35%), followed by caffeic acid derivative (16%), parishin G isomers (12%), and parishin B (7%). In terms of the total content of bioactive compounds, the methanol extract presented approximately two-fold concentration compared to the aqueous extract.

### 2.2. Enzyme Inhibition

Enzyme inhibitors have received due interest in the management of several diseases due to their role in pathophysiological mechanisms [16]. In fact, enzymes drive biological processes and as such have become the main strategy in drug design. Type 2 diabetes is a chronic disease, characterized by hyperglycemia. To control the progression of these diseases, dietary recommendations such as enzyme inhibitors could play a pivotal role. Acarbose, approved by the Food and Drug Administration for the treatment of type 2 diabetes in adults, competitively and reversibly inhibits pancreatic α-amylase and membrane-bound intestinal α-glucosidase. However, side effects associated to the use of acarbose, along with escalating prevalence of type 2 diabetes worldwide, are urging the scientific community to find safer alternatives. From Table 4, it is shown that the methanol extract of *A. pyramidalis* tubers was a more potent inhibitor of α-amylase (15.76 mg acarbose equivalent (ACAE)/g extract) and α-glucosidase (20.07 mg ACAE/g extract) compared with the water extract (5.23 mg ACAE/g extract, for both enzymes).

**Figure 2:**
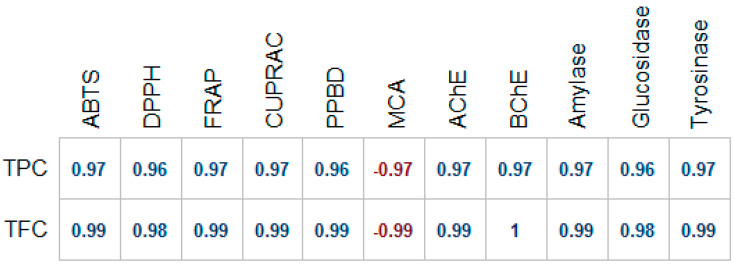
Relationship among total phenolic content (TPC), total flavonoid content (TFC), and biological activities (Pearson correlation coefficient, *p* < 0.05). PPBD: Phosphomolybdenum; MCA: Metal chelating assay.

The comorbidity of type 2 diabetes and Alzheimer disease has been supported by epidemiological evidences [17]. While referring to Alzheimer disease, researchers coined the term “type 3 diabetes” to underline the shared molecular and cellular features associated with insulin resistance, cognitive decline, and memory deficit [18]. Cholinesterase enzymes, responsible for the hydrolysis of the neurotransmitter acetylcholine, have been targeted in the management of Alzheimer disease. It has been observed that in the early stage of the disease a considerable increase in the activity of acetylcholinesterase was noted while butyrylcholinesterase activity, which has been often overlooked, shoots up to 90% in the late stages of Alzheimer disease, exacerbating the condition of the patient [19]. In the present study, the inhibitory capabilities of *A. pyramidalis* tubers’ extracts were assessed on both acetylcholinesterase and butyrylcholinesterase. As shown in Table 4, the water extract exhibited the lowest inhibitory action on acetylcholinesterase and butyrylcholinesterase. A higher inhibition was observed for the methanol extract; this could be attributed to the presence of caffeic acid or acacetin as well as the other unknown compounds [20,21]. Also, we observed a high correlation (R > 0.9) total bioactive compounds’ and enzyme inhibitory properties (Figure 2).

The increased demand for depigmenting products derived from natural products has been fueled by increased public concern towards synthetic agents. Tyrosinase inhibitors play a key role in the formulation of depigmenting products. In fact, the inhibition of tyrosinase, a copper-containing, rate-limiting enzyme responsible for the biosynthesis of melanin, has been directly related to the skin-lightening effect of dermatological and cosmetic products used for the management of epidermal hyperpigmentation conditions. The methanol extract (69.69 mg·kainic acid equivalents (KAE)/g extract) of *A. pyramidalis* tubers showed higher inhibition against tyrosinase compared with the water extract (11.09 mg·KAE/g extract). In silico molecular docking studies conducted by Pei and colleagues [22] reported that gastrodin, identified in a significant amount in the methanol extract of *A. pyramidalis* tubers, interacted primarily with histidine residues of tyrosinase active site. It was suggested that acacetin, identified *Agastache rugosa* Kuntze and *A. pyramidalis* tubers (Table 3), could be related to the observed tyrosinase inhibition [23].

### 2.3. Antioxidant Activity

Assessing antioxidant properties of plant extracts is crucial in the evaluation of plants’ bioactivity and plants’ ability to prevent and/or mitigate health problems. Investigations have demonstrated that intake of antioxidants could prevent or delay the onset/progress of several human ailments [24,25,26]. In order to obtain a comprehensive understanding of the antioxidant potential of *A. pyramidalis* tubers’ extracts, multiple antioxidant assays were employed. Results of the total antioxidant capacity, estimated using the phosphomolybdenum assay, are presented in Table 4. The methanol extract showed highest activity (0.73 mmol·trolox equivalents (TE)/g) for the phosphomolybdenum assay. A similar trend was observed for radical scavenging properties. Data shown in Table 5 revealed higher Trolox equivalent values for methanol extract, as regards the 2,2-diphenyl-1-picrylhydrazyl(DPPH) and 2,2′-azino-bis(3-ethylbenzothiazoline-6-sulfonic acid) (ABTS) radical scavenging assays. Likewise, a higher reducing activity was observed for the methanol extract in the CUPRAC and FRAP assays (45.56 and 29.38 mg·TE/g extract). The metal chelating properties of the extracts was also assessed. It was observed that the water extract exhibited high metal chelating potential. According to correlation analysis, a strong correlation between total phenolic, flavonoid content, and antioxidant properties (except for metal chelating) was observed (Figure 2). From this point, observed metal chelating activity could attribute to nonphenolic metal chelators in the extracts.

### 2.4. Prediction of Pharmacologic Targets and Pharmacokinetic Profile

**Figure 3:**
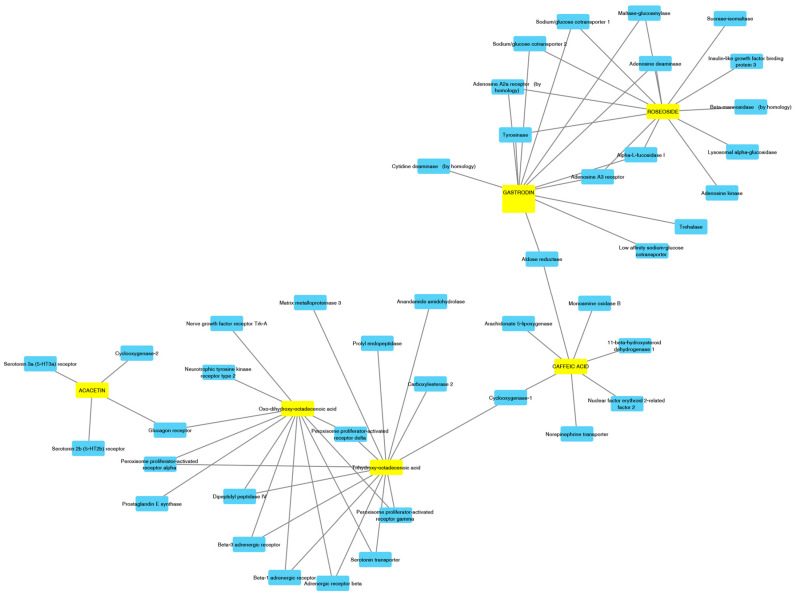
Pharmacological profile of phytocompounds identified through chromatographic analysis in methanol and water extracts of *A. pyramidalis* tubers. Molecular target and pharmacokinetic profile were predicted through SwissTargetPrediction (http://www.swisstargetprediction.ch/) and SwissADME (http://www.swissadme.ch/index.php) platforms, respectively. Finally, a component-target analysis was carried out through Cytoscape software (3.7.2 version) on acacetin, caffeic acid, dihydroxy-benzoic acid, gastrodin, oxo-dihydroxy-octadecenoic acid, parishin A, parishin B, parishin C, roseoside, and trihydroxy-octadecenoic acid. Extended results are included as Supplementary Materials (Supplementary Materials: Target proteins folder).

Based on qualitative phytochemical fingerprint assessment, a network pharmacology approach was conducted on selected extracts’ phytochemicals, in order to elucidate the putative target proteins underlying both the observed biological effects and the traditional uses. According to the pharmacokinetic predictions of gastrointestinal adsorption (results depicted in Supplementary Materials: Pharmacokinetic folder), carried out through SwissADME bioinformatic platform, acacetin, caffeic acid, dihydroxy-benzoic acid, gastrodin, oxo-dihydroxy-octadecenoic acid, parishin A, parishin B, parishin C, roseoside, and trihydroxy-octadecenoic acid were assayed for the identification of putative target proteins (SwissTargetPrediction bioinformatic platform). As expected, this last bioinformatic platform yielded a wide plethora of targets interacting with the selected secondary metabolites. Details about the bioinformatic analysis were reported in Supplementary Materials (target proteins folder), whereas component-target analysis, conducted with Cytoscape software, is illustrated in Figure 3. Specifically, component-target analysis indicated the capability of both roseoside and gastrodin to interact with tyrosinase. The putative interaction between gastrodin and tyrosinase was consistent with previous docking studies [23]. However, the sole roseoside could interact with glucosidase, whereas the interaction of the sole gastrodin with maltase glucoamylase was predicted, as well. Overall, these predicted interactions were consistent, at least in part, with the enzyme inhibitory effects induced by *A. pyramidalis* extracts, which could represent promising sources of bioactive compounds for the treatment of skin hyperpigmentation and type 2 diabetes. Regarding the putative antidiabetic activity of the tested extracts, it is also of noteworthy interest to highlight the interaction of oxo-dihydroxy-octadecenoic and trihydroxy-octadecenoic acid with dipeptidyl peptidase (DPP)-IV and peroxisome proliferator-activated receptor (PPAR)-α, which represent key targets of anti-diabetic therapy [27]. These compounds were also predicted to interact with multiple prostanoid receptors and enzymes, which could be at the basis of the efficacy of *A. pyramidalis* infusion against flu and cold [5]. Regarding the traditional uses of this plant against neurological and psychiatric diseases [4], some concerns arise from the network pharmacology approach on selected phytocompounds. On one side, caffeic acid, gastrodin, and acacetin were predicted to interact with target proteins involved in neurotransmitter pathways (i.e., dopamine, norepinephrine, serotonin, adenosine). On the other hand, the lack of any blood–brain barrier permeant property shown by selected compounds (depicted in Supplementary Materials: Pharmacokinetic folder) rule out a direct effect of *A. pyramidalis*-derived phytochemicals in central circuitries involved in neurological and neuropsychiatric effects. Nevertheless, considering the putative interactions with dipeptidyl peptidase IV, an enzyme deeply involved in the gut-brain axis mediated by neuropeptides [28], the present findings cannot exclude indirect effects induced by *A. pyramidalis* extracts on brain functions.

### 2.5. Docking Results

**Figure 4:**
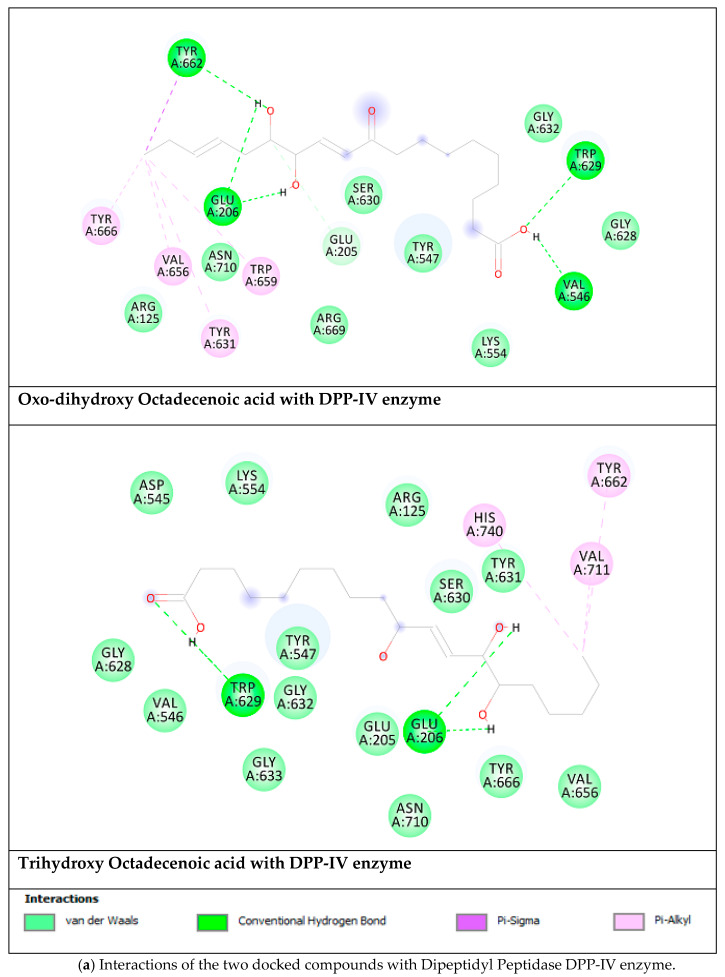

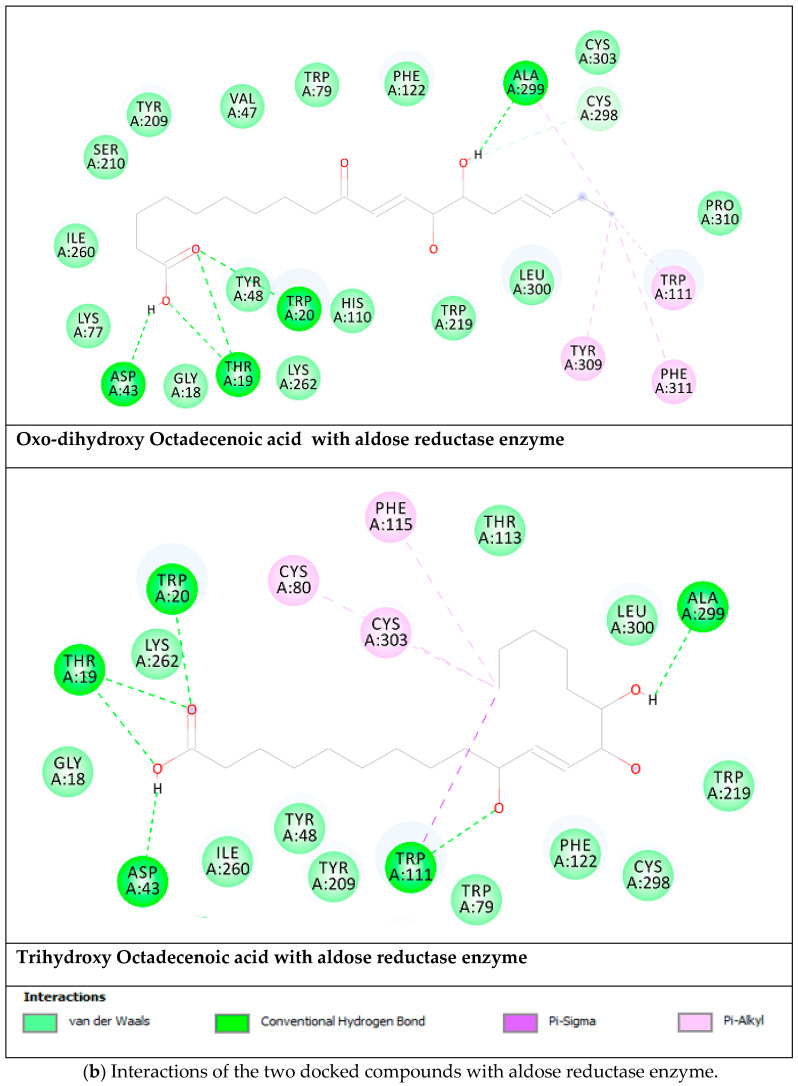

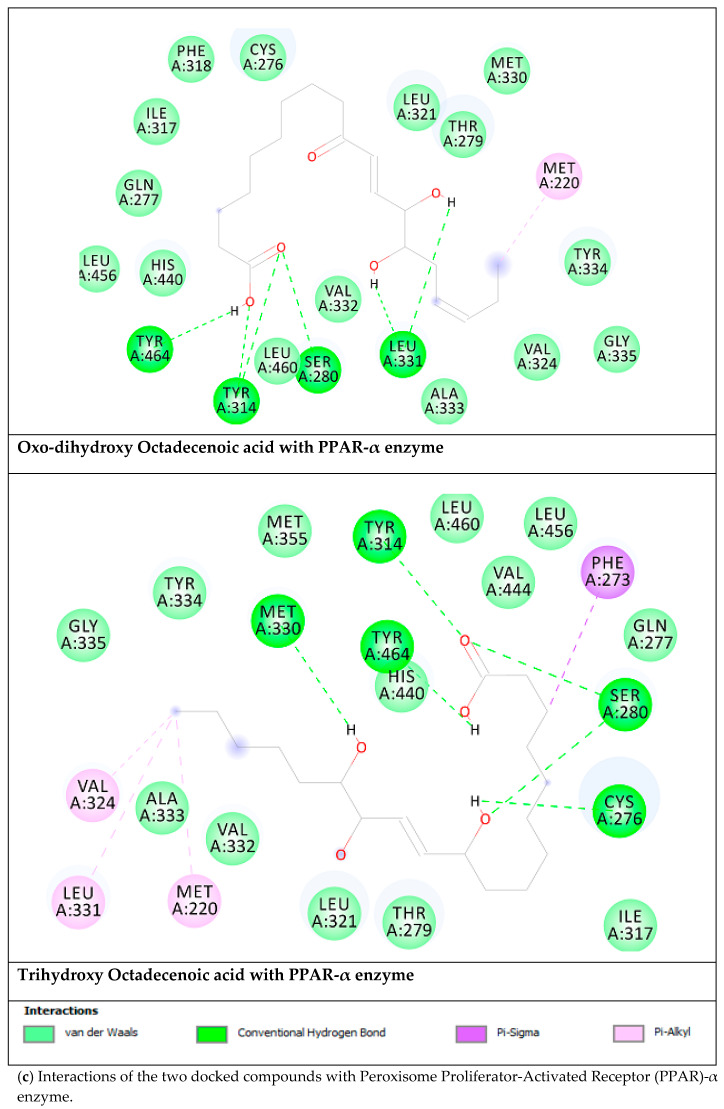

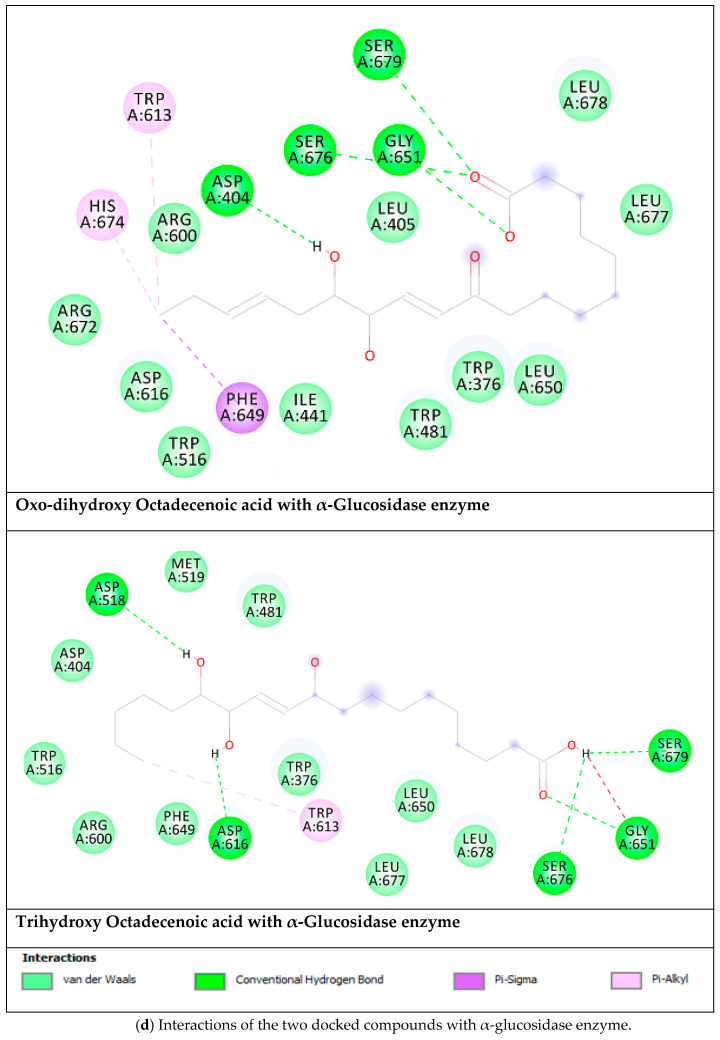
Nonbonding interactions of the docked compounds.

Based on the results of enzyme inhibition assays and network pharmacology analysis, a docking study was conducted to explore putative interactions between selected *A. pyramidalis* phytochemicals, namely oxo-dihydroxy octadecenoic acid and trihydroxy octadecenoic acid, and key enzymes involved in type 2 diabetes (i.e., aldose reductase, PPAR-α, DPP-IV, and α-glucosidase). The calculated binding free energy, inhibition constant (K_i_), and the nonbonding interactions represent the outcomes of any docking study. These parameters are essential to rank, compare, and design inhibitors for any drug design study. The results of docking study are listed in Table 6. Interestingly, oxo-dihydroxy octadecenoic acid showed higher binding affinity than trihydroxy octadecenoic acid toward the selected enzymes. In addition, both compounds showed the same trend in which the binding affinity went from aldose reductase, PPAR-α, DPP-IV, and α-glucosidase enzymes. Obviously, hydrogen bonds are the abundant interaction of these compounds, however, the number of these interactions are different for each compound and each enzyme. The hydrogen bond interactions and other hydrophobic interactions of the studied compounds are shown in Figure 4.

## 3. Materials and Methods

### 3.1. Plant Material and Preparation of Extracts

The plant materials were collected in Turkey (Kastamonu) in 2019 from wild, flowering plants. Taxonomical identification was performed by co-author, the botanist Dr. Ismail Senkardes. A voucher specimen is conserved at Marmara University, Faculty of Pharmacy Herbarium. Collection was performed to obtain representative samples without damaging the consistence of wild populations. The tubers were manually separated, cleaned, roughly sectioned, and taken to dryness in a ventilated oven (temperature 45 °C) until reaching a constant weight. The dry plant material was powdered using a laboratory mill and directly used for extraction or stored in a vacuo-plastic bag and stored in the dark until used.

Extraction by maceration was used to obtain methanol extract. Briefly, pulverized tuber (5 g) was stirred with 100 mL of methanol for 24 h at room temperature. The solution was then filtered and the solvent was evaporated by using rotary evaporator. Infusion was selected for water extract. Briefly, the plant material (5 g) was kept in the boiled water (100 mL) for 15 min. After cooling, the extract was filtered and lyophilized. The obtained dry extracts were stored at +4 °C in the dark.

### 3.2. Profile of Bioactive Compounds

To obtain total levels of phenolic and flavonoid content in the extracts, colorimetric assays were used as described in our previous paper [29]. Gallic acid ((GAE) for total phenolic (TPC)) and rutin ((RE) for total flavonoid (TFC)).

### 3.3. Chromatographic Analysis

For HPLC-MS analysis, 5 mg of dried extract (DE) were re-dissolved in 1 mL of methanol, filtered through 0.45 µm polytetrafluoroethylene (PTFE) membrane filters, and 10 μL of the solution was injected. The phytochemical profile was obtained with an Agilent Series 1100 liquid chromatograph with a G1315B diode array detector and an ion trap mass spectrometer (Esquire 6000, Bruker Daltonics, (Billerica, Massachusetts, USA) with an electrospray interface. A Luna Omega Polar C_18_ analytical column of 150 × 3.0 mm and 5 µm particle size (Phenomenex) was used. The method was adapted from a previous article of our research groups [30]. Separation was performed with a mobile phase of water-formic acid (100:0.1, *v/v*) and CH_3_CN. The following program was used: (1) Initial mobile phase, 10% CH_3_CN; (2) linear increase from 10% to 25% CH_3_CN (0–25 min); (3) 25% CH_3_CN (25–30 min); (4) linear increase from 25% to 50% CH_3_CN (30–40 min); (5) linear increase from 50% to 100% CH_3_CN (40–42 min); and (6) 100% CH_3_CN (42–47 min). Then, CH_3_CN percentage was returned to the initial mobile phase, with a 7 min stabilization time. The flow rate was 0.4 mL min^−1^. The scan range was *m/z* 100–1200 with a speed of 13,000 Da/s. The electrospray ionization (ESI) conditions were: Drying gas (N_2_) flow rate and temperature, 10 mL/min and 365 °C; nebulizer gas (N_2_) pressure, 50 psi; capillary voltage, 4500 V; capillary exit voltage, −117.3 V. We used the auto MS^n^ mode for the acquisition of MS^n^ data, with isolation width of 4.0 *m/z*, and fragmentation amplitude of 0.6 V (MS^n^ up to MS^4^).

### 3.4. Determination of Antioxidant and Enzyme Inhibitory Effects

The antioxidant potential of the extracts was evaluated by phosphomolybdenum, antiradical (DPPH and ABTS), reducing power (FRAP and CUPRAC) and ferrous chelating assays, as described by Grochowski et al. [31]. Trolox equivalents were used for expression of antioxidant activities. Ethylenediaminetetraacetic acid was employed as a reference compound for the metal chelating assay. The key enzymes’ inhibition activity of the extracts against ACh, BChE, tyrosinase, α-glucosidase, and α-amylase were measured as previously reported [31].

### 3.5. Prediction of Putative Targets and Pharmacokinetics

Putative targets were identified through the bioinformatic method recently described by Gu and colleagues [32]. Briefly, chemical structures were prepared and converted in canonical “Simplified Molecular Input Line Entry System” (SMILES) using ChemSketch software (12.0 version). The SMILES were then processed by the SwissTargetPrediction (http://www.swisstargetprediction.ch/) and SwissADME (http://www.swissadme.ch/index.php) platforms, for predicting putative targets and pharmacokinetic profile, respectively. The name of identified targets were normalized according to UniProt database (https://www.uniprot.org/). Finally, Cytoscape software (3.7.2 version) was used to create a component-target illustration network.

### 3.6. Docking Calculations

In order to investigate the binding affinity and the nonbonding interactions of the compounds, oxo-dihydroxy octadecenoic acid and trihydroxy octadecenoic acid against aldose reductase, DPP-IV, PPAR-α, and α-glucosidase enzymes, docking calculations were performed using Autodock4 software (version 4.2) (Molinspiration Database) [33]. The crystal structures of the target enzymes were downloaded from Protein Data Bank (PDB). PDBID:4GCA was used to get the crystal structure of aldose reductase enzyme in which the enzyme was crystalized with IDD 1219 inhibitor, PDBID:4N8D and PDBID:1K7L were used in the case of DPP-IV and PPAR-α enzymes, respectively, and PDBID:5NN5 for the crystal structure of α-glucosidase enzyme. All the co-crystalized molecules, such as the inhibitor and water molecules, were removed and all the proteins were neutralized by adding polar hydrogen atoms and Kollman united atom charges. The initial 3D structures of the two compounds were optimized using AM1 semi-empirical method [34] and the structures were saved in mol2 format. Autogrid 4 was used to design a grid box with 60 × 60 × 60 dimensions with 0.375 Å distance between points. Lamarckian genetic algorithm was used to scan 250 conformations for each inhibitor. The values of the calculated binding affinity were clustered and ranked in the output file. Discovery studio 5.0 visualizer was used to view the results and study the enzyme-inhibitor nonbonding interactions.

### 3.7. Statistical Analysis

The antioxidant and enzyme inhibitory activities results are given as mean ± standard deviation (S.D.). The results were statistically evaluated using the student t-test (α = 0.05). Pearson correlation coefficients were also calculated for total bioactive compounds and biological activities. Statistical analysis was carried out using SPSS v. 14.0 program (SPSS Inc, Chicago, IL, USA).

## 4. Conclusions

The current investigation highlighted the pharmacological potential of orchids, with a particular focus on traditionally used *A. pyramidalis* tubers. This study also supported the use of cutting-edge technologies to unravel the phytochemical profile of herbal extracts. The identification of phytochemicals could then sustain observed biological activities, with particular regards to enzyme inhibition. Parishin G isomer-2, gastrodin, and caffeic acid derivatives were the main compounds in the tested extracts and the extracts exhibited potent enzyme inhibitory, radical scavenging, and reducing properties. In addition, the outcomes of network pharmacology and docking calculations showed the affinity of oxo-dihydroxy octadecenoic acid and trihydroxy octadecenoic acid against aldose reductase, PPAR-α, DPP-IV, and α-glucosidase enzymes, which suggests the pharmacological potential of *A. pyramidalis* phytochemicals against type 2 diabetes.

## Figures and Tables

**Table 1:** Total bioactive components of the tested samples.

Extracts	Extraction Yield (%)	Total Phenolic Content (mg·gallic acid Equivalent (GAE)/g Extract)	Total Flavonoid Content (mg·rutin Equivalent (RE)/g Extract)
Methanol	13.24	17.03 ± 0.06 ^a^	0.48 ± 0.05 ^a^
Water	13.08	16.64 ± 0.06 ^a^	0.02 ± 0.01 ^b^

Values expressed are means ± S.D. of three parallel measurements. GAE: Gallic acid equivalent; RE: Rutin equivalent. Different letters (^a^ and ^b^) indicate significant differences in the extracts (*p* < 0.05).

**Table 2:** Characterization of the compounds found in the analyzed extracts of *Anacamptis pyramidalis*.

No.	t*_R_* (min)	[M − H]^−^ *m/z*	*m/z* (% Base Peak)	Assigned Identification	Methanol	Water
1	1.9	341	MS^2^ [341]: 179 (100), 161 (26), 149 (8), 143 (13), 131 (7), 119 (8), 113 (15)	Disaccharide	✓	✓
2	2.7	191	MS^2^ [191]: 173 (62), 111 (100)	Citric acid	✓	✓
3	4.7	459	MS^2^ [459]: 173 (100) MS^3^ [459→173]: 111 (100)	Parishin G isomer-1		✓
4	5.3	367	MS^2^ [367]: 293 (21), 187 (20), 143 (100) MS^3^ [367→143]: 125 (100)	Unknown	✓	
5	5.9	433	MS^2^ [433]: 227(7), 205 (100) MS^3^ [433→205]: 143 (62), 115 (100)	Unknown	✓	✓
6	6.1	459	MS^2^ [459]: 173 (100) MS^3^ [459→173]: 111 (100)	Parishin G isomer-2		✓
7	9.8	351	MS^2^ [351]: 171 (100), 127 (22) MS^3^ [351→171]: 127 (100)	Unknown	✓	✓
8	10.7	431	MS^2^ [431]: 385 (100), 223 (15) MS^3^ [431→385]: 223 (42), 153 (100), 138 (43)	Roseoside (formate adduct)	✓	
9	11.7	635	MS^2^ [635]: 349 (100), 277 (56) MS^3^ [635→349]: 305 (19), 277 (100), 169 (89), 143 (43)	Gastrodin derivative	✓	
10	12.4	727	MS^2^ [727]: 459 (4), 441 (38), 423 (100), 397 (21), 369 (18), 263 (3)	Parishin B		✓
11	12.8	473	MS^2^ [473]: 285 (100), 187 (27), 159 (53), 143 (63) MS^3^ [473→285]: 161 (39), 123 (100)	Gastrodin derivative	✓	✓
12	13.9	635	MS^2^ [635]: 349 (100), 277 (38) MS^3^ [635→349]: 277 (100), 169 (66), 143 (50)	Gastrodin derivative	✓	
13	14.1	727	MS^2^ [727]: 459 (3), 441 (46), 423 (100), 397 (26), 369 (25), 263 (5)	Parishin C		✓
14	16.5	473	MS^2^ [473]: 285 (100), 187 (11), 169 (20), 159 (36), 143 (48) MS^3^ [473→285]: 161 (23), 123 (100)	Gastrodin derivative	✓	✓
15	17.7	619	MS^2^ [619]: 439 (100) MS^3^ [619→439]: 171 (32), 153 (100) MS^4^ [619→439→153]: 138 (100), 109 (45)	Dihydroxybenzoic acid derivative	✓	
16	18.6	995	MS^2^ [995]: 727 (100) MS^3^ [995→727]: 459 (7), 441 (28), 423 (100), 397 (27), 369 (18), 263 (1)	Parishin		✓
17	19.9	741	MS^2^ [741]: 473 (100) MS^3^ [741→473]: 285 (100), 187 (13), 159 (24), 143 (62) MS^4^ [741→473→285]: 161 (65), 123 (100)	Gastrodin derivative	✓	✓
18	22.3	887	MS^2^ [887]: 619 (100), 439 (32) MS^3^ [887→619]: 439 (100) MS^4^ [887→619→439]: 171 (33), 153 (100)	Unknown	✓	
19	23.3	457	MS^2^ [457]: 153 (100) MS^3^ [457→153]: 109 (100)	Dihydroxybenzoic acid derivative	✓	✓
20	29.0	725	MS^2^ [725]: 457 (100) MS^3^ [725→457]: 285 (39), 153 (100) MS^4^ [725→457→153]: 109 (100)	Dihydroxybenzoic acid derivative	✓	✓
21	30.6	282	MS^2^ [282]: 145 (100), 119 (73)	Unknown	✓	✓
22	32.0	312	MS^2^ [312]: 179 (61), 135 (100)	Caffeic acid derivative	✓	✓
23	38.2	623	MS^2^ [623]: 461 (100), 283 (38) MS^3^ [623→461]: 283 (100) MS^4^ [623→461→283]: 268 (100)	Acacetin derivative	✓	✓
24	38.8	623	MS^2^ [623]: 461 (100) MS^3^ [623→461]: 283 (100) MS^4^ [623→461→283]: 268 (100)	Acacetin derivative	✓	✓
25	39.2	327	MS^2^ [327]: 309 (27), 291 (55), 229 (48), 211 (48), 171 (100)	Oxo-dihydroxy-octadecenoic acid	✓	
26	40.6	329	MS^2^ [329]: 311 (47), 229 (72), 211 (56), 171 (100)	Trihydroxy-octadecenoic acid	✓	

**Table 3:** Relative peak areas and heat map obtained by HPLC coupled to mass spectrometry (MS^n^)-electrospray ionization (ESI) analysis of extracts of *A. pyramidalis*.

Peak	Compound	Methanol	Water
1	Disaccharide	2.02	1.83
2	Citric acid	0.14	1.65
3	Parishin G isomer-1	0.00	1.11
4	Unknown	6.50	0.00
5	Unknown	0.80	1.08
6	Parishin G isomer-2	0.00	10.96
7	Unknown	10.03	0.48
8	Roseoside	0.16	0.00
9	Gastrodin derivative	4.35	0.00
10	Parishin B	0.00	7.25
11	Gastrodin derivative	5.14	6.28
12	Gastrodin derivative	1.41	0.00
13	Parishin C	0.00	1.53
14	Gastrodin derivative	9.58	16.03
15	Dihydroxybenzoic acid derivative	5.04	0.00
16	Parishin	0.00	5.90
17	Gastrodin derivative	18.01	13.34
18	Unknown	3.19	0.00
19	Dihydroxybenzoic acid derivative	2.75	6.11
20	Dihydroxybenzoic acid derivative	3.57	3.46
21	Unknown	2.28	1.73
22	Caffeic acid derivative	15.94	16.20
23	Acacetin derivative	2.76	2.85
24	Acacetin derivative	3.23	2.20
25	Oxo-dihydroxy-octadecenoic acid	0.19	0.00
26	Trihydroxy-octadecenoic acid	2.91	0.00

**Table 4:** Enzyme inhibitory properties of the tested extracts.

Extracts	AChE (mg·GALAE/g Extract)	BChE (mg·GALAE/g Extract)	Tyrosinase (mg·KAE/g Extract)	α-Amylase (mg·ACAE/g Extract)	α-Glucosidase (mg·ACAE/g Extract)
Methanol	0.97 ± 0.01 ^a^	0.78 ± 0.03 ^a^	69.69 ± 0.29 ^a^	15.76 ± 0.25 ^a^	20.07 ± 4.29 ^a^
Water	0.04 ± 0.01 ^b^	0.18 ± 0.02 ^b^	11.09 ± 1.40 ^b^	5.23 ± 0.10 ^b^	5.23 ± 0.40 ^b^

Values expressed are means ± S.D. of three parallel measurements. GALAE: Galantamine equivalent; KAE: Kojic acid equivalent; ACAE: Acarbose equivalent; na: Not active. Different letters (^a^ and ^b^) indicate significant differences in the extracts (*p* < 0.05).

**Table 5:** Antioxidant activities of the tested samples.

Extracts	Phosphomolybdenum (mmol·TE/g)	DPPH (mg·TE/g Extract)	ABTS (mg·TE/g Extract)	CUPRAC (mg·TE/g Extract)	FRAP (mg·TE/g Extract)	Metal Chelating Ability (mg·EDTAE/g)
Methanol	0.73 ± 0.03 ^a^	22.12 ± 0.69 ^a^	44.23 ± 0.29 ^a^	45.56 ± 0.81 ^a^	29.38 ± 0.57 ^a^	11.10 ± 0.44 ^b^
Water	0.42 ± 0.02 ^b^	9.73 ± 0.11 ^b^	29.83 ± 0.63 ^b^	26.99 ± 0.19 ^b^	21.70 ± 0.25 ^b^	21.14 ± 0.44 ^a^

Values expressed are means ± S.D. of three parallel measurements. TE: Trolox equivalent; EDTAE: Ethylenediaminetetraacetic acid (EDTA) equivalent. Different letters (^a^ and ^b^) indicate significant differences in the extracts (*p* < 0.05).

**Table 6:** The calculated binding free energy, ∆G, in kcal/mol, inhibition constant, K_i_, the key residues and the number of hydrogen atoms of the docked compounds.

Targets	∆G (K_i_)	Key Residues	no. of HB
**Oxo-Dihydroxy Octadecenoic acid**			
Aldose reductase	−9.92 (53.7 nM)	Ala299(HB), Trp20 (HB), Thr19 (HB), Asp43(HB), Trp111, Phe311, Tyr309	**4**
DPP-IV	−6.06 (35.9 µM)	Tyr662 (HB), Trp629 (HB), Val546 (HB), Glu206 (HB), Trp659, Tyr631, Val656, Tyr666.	**5**
PPAR-α	−7.04 (6.9 µM)	Tyr464 (HB), Tyr314 (HB), Ser280 (HB), Leu331 (HB), Met220	**6**
α-Glucosidase	−6.06 (36.1 µM)	Ser679 (HB), Gly651 (HB), Ser676 (HB), Asp404 (HB), Trp613, His674, Phe649	**4**
**Trihydroxy Octadecenoic acid**			
Aldose reductase	−9.08 (222.3 nM)	Ala299 (HB), Trp111 (HB), Asp43 (HB),Thr9 (HB),Trp20 (HB), Cys303, Cys80, Phe115	**6**
DPP-IV	−5.82 (54.2 µM)	Trp629 (HB), Glu206 (HB), His740, Val711, Tyr662.	**3**
PPAR-α	−6.74 (11.4 µM)	Met330 (HB), Tyr314 (HB), Tyr464 (HB), Ser280 (HB), Cys276 (HB), Phe273, Met220, Leu331, Val324	**6**
α-Glucosidase	−4.63 (400.5 µM)	Asp518 (HB), Asp616 (HB), Ser679 (HB), Gly651 (HB), Ser676 (HB), Trp613	**5**

## Supplementary Materials

The supplementary material and methods are available online at https://www.mdpi.com/1420-3049/25/10/2422/s1.

## References

[1] Hao D.C., Xiao P.G. (2020). Pharmaceutical resource discovery from traditional medicinal plants: Pharmacophylogeny and pharmacophylogenomics. Chin. Herb. Med..

[2] Chinsamy M., Finnie J.F., Van Staden J. (2011). The ethnobotany of South African medicinal orchids. South Afr. J. Bot..

[3] Bhattacharyya P., Van Staden J. (2016). Ansellia africana (Leopard orchid): A medicinal orchid species with untapped reserves of important biomolecules—A mini review. South Afr. J. Bot..

[4] Bhattacharyya P., Kumaria S., Tandon P. (2015). Applicability of ISSR and DAMD markers for phyto-molecular characterization and association with some important biochemical traits of Dendrobium nobile, an endangered medicinal orchid. Phytochemistry.

[5] Sargin S.A., Büyükcengiz M. (2019). Plants used in ethnomedicinal practices in Gulnar district of Mersin, Turkey. J. Herb. Med..

[6] Sargin S.A., Selvi S., Büyükcengiz M. (2015). Ethnomedicinal plants of Aydıncık District of Mersin, Turkey. J. Ethnopharmacol..

[7] Tekinşen K.K., Güner A. (2010). Chemical composition and physicochemical properties of tubera salep produced from some Orchidaceae species. Food Chem..

[8] Ghorbani A., Gravendeel B., Naghibi F., de Boer H. (2014). Wild orchid tuber collection in Iran: A wake-up call for conservation. Biodivers. Conserv..

[9] Masters S., van Andel T., de Boer H.J., Heijungs R., Gravendeel B. (2020). Patent analysis as a novel method for exploring commercial interest in wild harvested species. Biol. Conserv..

[10] Swarts N.D., Dixon K.W. (2009). Terrestrial orchid conservation in the age of extinction. Ann. Bot..

[11] Kreziou A., de Boer H., Gravendeel B. (2016). Harvesting of salep orchids in north-western Greece continues to threaten natural populations. Oryx.

[12] Wang L., Xiao H.B., Yang L., Wang Z.T. (2012). Two new phenolic glycosides from the rhizome of Gastrodia elata. J. Asian Nat. Prod. Res..

[13] Wang L., Xiao H., Liang X., Wei L. (2007). Identification of phenolics and nucleoside derivatives in Gastrodia elata by HPLC-UV-MS. J. Sep. Sci..

[14] Spínola V., Llorent-Martínez E.J., Gouveia S., Castilho P.C. (2014). Myrica faya: A new source of antioxidant phytochemicals. J. Agric. Food Chem..

[15] Van Hoyweghen L., De Bosscher K., Haegeman G., Deforce D., Heyerick A. (2014). In vitro inhibition of the transcription factor NF-κB and cyclooxygenase by Bamboo extracts. Phytother. Res..

[16] Pattan S., Shirote P., Pattan J., Manvi F.V. (2008). Treatment of chronic diseases through enzyme inhibition. Indian Drugs.

[17] Barbagallo M., Dominguez L.J. (2014). Type 2 diabetes mellitus and Alzheimer’s disease. World J. Diabetes.

[18] Kandimalla R., Thirumala V., Reddy P.H. (2017). Is Alzheimer’s disease a Type 3 Diabetes? A critical appraisal. Biochim. Biophys. Acta (BBA)-Mol. Basis Dis..

[19] Darvesh S. (2016). Butyrylcholinesterase as a diagnostic and therapeutic target for Alzheimer’s disease. Curr. Alzheimer Res..

[20] Agunloye O.M., Oboh G. (2017). Modulatory effect of caffeic acid on cholinesterases inhibitory properties of donepezil. J. Complement. Integr. Med..

[21] Nugroho A., Park J.H., Choi J.S., Park K.S., Hong J.P., Park H.J. (2019). Structure determination and quantification of a new flavone glycoside with anti-acetylcholinesterase activity from the herbs of Elsholtzia ciliata. Nat. Prod. Res..

[22] Pei C.J., Lee J., Si Y.X., Oh S., Xu W.A., Yin S.J., Qian G.Y., Han H.Y. (2013). Inhibition of tyrosinase by gastrodin: An integrated kinetic-computational simulation analysis. Process. Biochem..

[23] Kim N.Y., Kwon H.S., Lee H.Y. (2017). Effect of inhibition on tyrosinase and melanogenesis of Agastache rugosa Kuntze by lactic acid bacteria fermentation. J. Cosmet. Dermatol..

[24] Feng Y., Wang X. (2012). Antioxidant therapies for Alzheimer’s disease. Oxid Med. Cell Longev..

[25] Hajhashemi V., Vaseghi G., Pourfarzam M., Abdollahi A. (2010). Are antioxidants helpful for disease prevention?. Res. Pharm. Sci..

[26] Young I.S., Woodside J.V. (2001). Antioxidants in health and disease. J. Clin. Pathol..

[27] Aroor A.R., Manrique-Acevedo C., DeMarco V.G. (2018). The role of dipeptidylpeptidase-4 inhibitors in management of cardiovascular disease in diabetes; focus on linagliptin. Cardiovasc Diabetol..

[28] Valassi E., Scacchi M., Cavagnini F. (2008). Neuroendocrine control of food intake. Nutr. Metab. Cardiovasc Dis..

[29] Chiavaroli A., Recinella L., Ferrante C., Locatelli M., Macchione N., Zengin G., Leporini L., Leone S., Martinotti S., Brunetti L. (2017). Crocus sativus, Serenoa repens and Pinus massoniana extracts modulate inflammatory response in isolated rat prostate challenged with LPS. J. Boil. Regul. Homeost. Agents.

[30] Llorent-Martínez E.J., Zengin G., Lobine D., Molina-García L., Mollica A., Mahomoodally M.F. (2018). Phytochemical characterization, in vitro and in silico approaches for three Hypericum species. New J. Chem..

[31] Zengin G., Locatelli M., Stefanucci A., Macedonio G., Novellino E., Mirzaie S., Dvorácskó S., Carradori S., Brunetti L., Orlando G. (2017). Chemical characterization, antioxidant properties, anti-inflammatory activity, and enzyme inhibition of Ipomoea batatas L. leaf extracts. Int. J. Food Prop..

[32] Gu L., Lu J., Li Q., Wu N., Zhang L., Li H., Xing W., Zhang X. (2020). A network-based analysis of key pharmacological pathways of Andrographis paniculata acting on Alzheimer’s disease and experimental validation. J. Ethnopharmacol..

[33] The Molinspiration Database. http://www.molinspiration.com.

[34] Frisch M., Trucks G., Schlegel H., Scuseria G., Robb M., Cheeseman J., Scalmani G., Barone V., Mennucci B., Petersson G. (2009). Gaussian 09.

