# Supplementary material for: Phytochemical Analysis, Network Pharmacology and in Silico Investigations on Anacamptis pyramidalis Tuber Extracts
Source: Molecules. 2020 May 22;25(10):2422. doi: 10.3390/molecules25102422 (PMC7288046; doi:10.3390/molecules25102422)
Supplement: Supplementary file 1 [file molecules-25-02422-s001.zip › Supplementary Materials - 2/Scavenger-Reducing-Enzyme Inhibition.pdf]

Article

Phytochemical analysis, network pharmacology and *in silico* investigations on *Anacamptis pyramidalis* tuber extracts

Mohamad Fawzi Mahomoodally[1,2], Marie Carene Nancy Picot-Allain[3], Gokhan Zengin[4*], Eulogio J. Llorent-Martínez [5], Hassan H. Abdullah[6], Gunes Ak[4], Ismail Senkardes[7], Annalisa Chiavaroli[8], Luigi Menghini[8*], Lucia Recinella[8], Luigi Brunetti[8], Sheila Leone[8], Giustino Orlando[8] and Claudio Ferrante[8]

[1]Department for Management of Science and Technology Development, Ton Duc Thang University, Ho Chi Minh City, Vietnam; mohamad.fawzi.mahomoodally@tdtu.edu.vn (M.F.M)

[2]Faculty of Applied Sciences, Ton Duc Thang University, Ho Chi Minh City, Vietnam; mohamad.fawzi.mahomoodally@tdtu.edu.vn (M.F.M)

[3]Department of Health Sciences, Faculty of Science, University of Mauritius, 230 Réduit, Mauritius; picotcarene@yahoo.com (M.C.N.P)

[4]Department of Biology, Science Faculty, Selcuk University, Campus, 42130, Konya, Turkey; gokhanzengin@selcuk.edu.tr (GZ), akguneselcuk@gmail.com (GA)

[5]Department of Physical and Analytical Chemistry, University of Jaén, Campus Las Lagunillas S/N, E-23071 Jaén, Spain; ellorent@ujaen.es (E.J.L)

[6]Chemistry department, College of Education, Salahaddin University-Erbil, 44001, Erbil, Iraq. e-mail; hassan.abdallah@su.edu.krd

[7]Department of Pharmaceutical Botany, Faculty of Pharmacy, Marmara University, Istanbul, Turkey; isenkardes@marmara.edu.tr

[8]Department of Pharmacy, "G. d'Annunzio" University Chieti-Pescara, 66100 Chieti, Italy; annalisa.chiavaroli@unich.it (A.C.); luigi.menghini@unich.it (L.M.); lucia.recinella@unich.it (L.R.); luigi.brunetti@unich.it (L.B.); sheila.leone@unich.it (S.L.); claudio.ferrante@unich.it (C.F.)

[*] Corresponding authors: Dr Gokhan Zengin (gokhanzengin@selcuk.edu.tr); Prof. Luigi Menghini (luigi.menghini@unich.it)

Assays for Total Phenolic and Flavonoid Contents

The total phenolic content was determined by employing the methods given in the literature with some modification. Sample solution (0.25 mL) was mixed with diluted Folin–Ciocalteu reagent (1 mL, 1:9, v/v) and shaken vigorously. After 3 min, Na_2CO_3 solution (0.75 mL, 1%) was added and the sample absorbance was read at 760 nm after a 2 h incubation at room temperature. The total phenolic content was expressed as milligrams of gallic acid equivalents (mg GAE/g extract)[1].

The total flavonoid content was determined using the $AlCl_3$ method. Briefly, sample solution (1 mL) was mixed with the same volume of aluminum trichloride (2%) in methanol. Similarly, a blank was prepared by adding sample solution (1 mL) to methanol (1 mL) without $AlCl_3$. The sample and blank absorbances were read at 415 nm after a 10 min incubation at room temperature. The absorbance of the blank was subtracted from that of the sample. Rutin was used as a reference standard and the total flavonoid content was expressed as milligrams of rutin equivalents (mg RE/g extract) [1].

Determination of Antioxidant and Enzyme Inhibitory Effects

Antioxidant (DPPH and ABTS radical scavenging, reducing power (CUPRAC and FRAP), phosphomolybdenum and metal chelating (ferrozine method)) and enzyme inhibitory activities (cholinesterase (Elmann's method), tyrosinase (dopachrome method), α-amylase (iodine/potassium iodide method), α -glucosidase (chromogenic PNPG method) and pancreatic lipase (*p*-nitrophenyl butyrate (p-NPB) method) were determined using the methods previously described by Uysal et al. [1] and Grochowski et al. [2]

For the DPPH (1,1-diphenyl-2-picrylhydrazyl) radical scavenging assay: Sample solution was added to 4 mL of a 0.004% methanol solution of DPPH. The sample absorbance was read at 517 nm after a 30 min incubation at room temperature in the dark. DPPH radical scavenging activity was expressed as milligrams of trolox equivalents (mg TE/g extract).

For ABTS (2,2′-azino-bis(3-ethylbenzothiazoline) 6-sulfonic acid) radical scavenging assay: Briefly, ABTS+ was produced directly by reacting 7 mM ABTS solution with 2.45 mM potassium persulfate and allowing the mixture to stand for 12–16 in the dark at room temperature. Prior to beginning the assay, ABTS solution was diluted with methanol to an absorbance of 0.700 ± 0.02 at 734 nm. Sample solution was added to ABTS solution (2 mL) and mixed. The sample absorbance was read at 734 nm after a 30 min incubation at room temperature. The ABTS radical scavenging activity was expressed as milligrams of trolox equivalents (mg TE/g extract).

For CUPRAC (cupric ion reducing activity) activity assay: Sample solution was added to premixed reaction mixture containing $CuCl_2$ (1 mL, 10 mM), neocuproine (1

mL, 7.5 mM) and NH_4Ac buffer (1 mL, 1 M, pH 7.0). Similarly, a blank was prepared by adding sample solution (0.5 mL) to premixed reaction mixture (3 mL) without $CuCl_2$. Then, the sample and blank absorbances were read at 450 nm after a 30 min incubation at room temperature. The absorbance of the blank was subtracted from that of the sample. CUPRAC activity was expressed as milligrams of trolox equivalents (mg TE/g extract).

For FRAP (ferric reducing antioxidant power) activity assay: Sample solution was added to premixed FRAP reagent (2 mL) containing acetate buffer (0.3 M, pH 3.6), 2,4,6-tris(2-pyridyl)-S-triazine (TPTZ) (10 mM) in 40 mM HCl and ferric chloride (20 mM) in a ratio of 10:1:1 (v/v/v). Then, the sample absorbance was read at 593 nm after a 30 min incubation at room temperature. FRAP activity was expressed as milligrams of trolox equivalents (mg TE/g extract).

For phosphomolybdenum method: Sample solution was combined with 3 mL of reagent solution (0.6 M sulfuric acid, 28 mM sodium phosphate and 4 mM ammonium molybdate). The sample absorbance was read at 695 nm after a 90 min incubation at 95 °C. The total antioxidant capacity was expressed as millimoles of trolox equivalents (mmol TE/g extract).

For metal chelating activity assay: Briefly, sample solution was added to $FeCl_2$ solution (0.05 mL, 2 mM). The reaction was initiated by the addition of 5 mM ferrozine (0.2 mL). Similarly, a blank was prepared by adding sample solution (2 mL) to $FeCl_2$ solution (0.05 mL, 2 mM) and water (0.2 mL) without ferrozine. Then, the sample and blank absorbances were read at 562 nm after 10 min incubation at room temperature. The absorbance of the blank was sub-tracted from that of the sample. The metal

chelating activity was expressed as milligrams of EDTA (disodium edetate) equivalents (mg EDTAE/g extract).

For Cholinesterase (ChE) inhibitory activity assay: Sample solution (was mixed with DTNB (5,5-dithio-bis(2-nitrobenzoic) acid, Sigma, St. Louis, MO, USA) (125 μL) and AChE (acetylcholines-terase (Electric ell acetylcholinesterase, Type-VI-S, EC 3.1.1.7,Sigma)), or BChE (butyrylcholinesterase (horse serum butyrylcholinesterase, EC 3.1.1.8, Sigma)) solution (25 μL) in Tris–HCl buffer (pH 8.0) in a 96-well microplate and incubated for 15 min at 25 °C. The reaction was then initiated with the addition of acetylthiocholine iodide (ATCI, Sigma) or butyrylthiocholine chloride (BTCl, Sigma) (25 μL). Similarly, a blank was prepared by adding sample solution to all reaction reagents without enzyme (AChE or BChE) solution. The sample and blank absorbances were read at 405 nm after 10 min incubation at 25 °C. The absorbance of the blank was subtracted from that of the sample and the cholinesterase inhibitory activity was expressed as galanthamine equivalents (mgGALAE/g extract).

For Tyrosinase inhibitory activity assay: Sample solution was mixed with tyrosinase solution (40 μL, Sigma) and phosphate buffer (100 μL, pH 6.8) in a 96-well microplate and incubated for 15 min at 25 °C. The reaction was then initiated with the addition of L-DOPA (40 μL, Sigma). Similarly, a blank was prepared by adding sample solution to all reaction reagents without enzyme (tyrosinase) solution. The sample and blank absorbances were read at 492 nm after a 10 min incubation at 25 °C. The absorbance of the blank was subtracted from that of the sample and the tyrosinase inhibitory activity was expressed as kojic acid equivalents (mgKAE/g extract).

For α-amylase inhibitory activity assay: Sample solution was mixed with α-amylase solution (ex-porcine pancreas, EC 3.2.1.1, Sigma) (50 μL) in phosphate buffer

(pH 6.9 with 6 mM sodium chloride) in a 96-well microplate and incubated for 10 min at 37 °C. After pre-incubation, the reaction was initiated with the addition of starch solution (50 μL, 0.05%). Similarly, a blank was prepared by adding sample solution to all reaction reagents without enzyme (α-amylase) solution. The reaction mixture was incubated 10 min at 37 °C. The reaction was then stopped with the addition of HCl (25 μL, 1 M). This was followed by addition of the iodine-potassium iodide solution (100 μL). The sample and blank absorbances were read at 630 nm. The absorbance of the blank was subtracted from that of the sample and the α-amylase inhibitory activity was expressed as acarbose equivalents (mmol ACE/g extract).

For α-glucosidase inhibitory activity assay: Sample solution was mixed with glutathione (50 μL), α-glucosidase solution (from Saccharomyces cerevisiae, EC 3.2.1.20, Sigma) (50 μL) in phosphate buffer (pH 6.8) and PNPG (4-N-trophenyl-α-D-glucopyranoside, Sigma) (50 μL) in a 96-well microplate and incubated for 15 min at 37 °C. Similarly, a blank was prepared by adding sample solution to all reaction reagents without enzyme (α-glucosidase) solution. The reaction was then stopped with the addition of sodium carbonate (50 μL, 0.2 M). The sample and blank absorbances were read at 400 nm. The absorbance of the blank was subtracted from that of the sample and the α-glucosidase inhibitory activity was expressed as acarbose equivalents (mmol ACE/g extract).

1. Uysal, S.; Zengin, G.; Locatelli, M.; Bahadori, M. B.; Mocan, A.; Bellagamba, G.; De Luca, E.; Mollica, A.; Aktumsek, A., Cytotoxic and enzyme inhibitory potential of two Potentilla species (P. speciosa L. and P. reptans Willd.) and their chemical composition. *Frontiers in pharmacology* **2017,** 8, 290.
2. Grochowski, D. M.; Uysal, S.; Aktumsek, A.; Granica, S.; Zengin, G.; Ceylan, R.; Locatelli, M.; Tomczyk, M., In vitro enzyme inhibitory properties, antioxidant activities, and phytochemical profile of Potentilla thuringiaca. *Phytochemistry Letters* **2017,** 20, 365-372.
